# Insights into the Molecular Evolution of the PDZ/LIM Family and Identification of a Novel Conserved Protein Motif

**DOI:** 10.1371/journal.pone.0000189

**Published:** 2007-02-07

**Authors:** Aartjan J.W. te Velthuis, Tadamoto Isogai, Lieke Gerrits, Christoph P. Bagowski

**Affiliations:** 1 Department of Integrative Zoology, Institute of Biology, Leiden University, Leiden, The Netherlands; 2 Department of Molecular and Cellular Biology, Institute of Biology, Leiden University, Leiden, The Netherlands; Wellcome Trust Centre for Human Genetics, United Kingdom

## Abstract

The PDZ and LIM domain-containing protein family is encoded by a diverse group of genes whose phylogeny has currently not been analyzed. In mammals, ten genes are found that encode both a PDZ- and one or several LIM-domains. These genes are: ALP, RIL, Elfin (CLP36), Mystique, Enigma (LMP-1), Enigma homologue (ENH), ZASP (Cypher, Oracle), LMO7 and the two LIM domain kinases (LIMK1 and LIMK2). As conventional alignment and phylogenetic procedures of full-length sequences fell short of elucidating the evolutionary history of these genes, we started to analyze the PDZ and LIM domain sequences themselves. Using information from most sequenced eukaryotic lineages, our phylogenetic analysis is based on full-length cDNA-, EST-derived- and genomic- PDZ and LIM domain sequences of over 25 species, ranging from yeast to humans. Plant and protozoan homologs were not found. Our phylogenetic analysis identifies a number of domain duplication and rearrangement events, and shows a single convergent event during evolution of the PDZ/LIM family. Further, we describe the separation of the ALP and Enigma subfamilies in lower vertebrates and identify a novel consensus motif, which we call ‘ALP-like motif’ (AM). This motif is highly-conserved between ALP subfamily proteins of diverse organisms. We used here a combinatorial approach to define the relation of the PDZ and LIM domain encoding genes and to reconstruct their phylogeny. This analysis allowed us to classify the PDZ/LIM family and to suggest a meaningful model for the molecular evolution of the diverse gene architectures found in this multi-domain family.

## Introduction

The sequencing and annotation of an increasing number of genomes has led to a huge amount of protein sequence data. The goal of functional genomics is to determine the function of these proteins. For this purpose, it is essential to construct a comprehensive evolutionary classification of proteins and their families, which can be especially useful if members of the same protein family have similar or identical biochemical functions [1]. The classification of protein families is based on homologous relationships and several methods are currently available for clustering proteins into families [2], [3]. Most of those approaches rely on sequence similarity measures, such as those obtained with BLAST [4] or hidden Markov models [5]. Because many proteins contain multiple domains, many of these methods of protein clustering result in the establishment of incorrect families. This problem is complicated in metazoan proteomes, and the human proteome in particular, where multi-domain proteins are abundant.

Domains are the building blocks of all globular proteins and present one of the most useful levels at which protein function can be understood [3]. There is a limited repertoire of types of domains [6], [7] and the domains from this set are duplicated and recombined in different ways to form the respective proteomes of various genomes in life. Although the presence of a shared domain (or more than one shared domain) can be an indicator of similar functions [8], it does not necessarily imply it [9]. The repertoire of different architectures present in the genomes has arisen by the duplication and recombination of the ancestral superfamily domains. Convergent evolution of gene architectures has been defined as more than one independent evolutionary event (recombination) leading to the same domain architecture [10].

PDZ and LIM domains are both interaction modules, present in proteins with diverse functions and assorted additional domains. Originally PDZ domains were recognized in the postsynaptic density protein PSD-95 [11], the septate junction protein Discs-large of *Drosophila melanogaster* [12] and the epithelial tight junction protein ZO-1 [13]. PDZ domains play important roles in organizing cell signaling assemblies [14] and are found in plants, yeast, bacteria and a variety of metazoans [15], [16]. They recognize short C-terminal peptide motifs, internal sequences resembling a C-terminus and have further been shown to bind to phospholipids [reviewed in 14], [17].

The predominance of PDZ domains in metazoans was proposed to indicate their co-evolution with multicellularity. Proportionately fewer PDZ domains are found in bacteria and yeast. However, a relatively low number of PDZ domains are found to be encoded in plant genomes. PDZ domains were found to be present in proteins from phylogenetically diverse groups of bacteria [18] and it was suggested that PDZ domains might have entered the bacterial and plant genomes by horizontal gene transfer. This hypothesis was based on the observation that human and bacterial htrA genes were significantly more similar to each other than either is to each of the yeast htra-like repeats [16]. Indeed, the yeast PDZ-like domains found in the four htrAs exhibit extremely low sequence homology to the metazoan consensus PDZ domains.

The LIM domain is a tandem zinc-finger structure that functions, like the PDZ, as a protein-protein interaction module [19]–[21]. LIM domains are found in proteins from a wide variety of eukaryotic organisms although fewer LIMs are found in yeast and plants compared to vertebrates for example [19], [22] (this is similar to the PDZ domains). We have found only a single bacterial LIM domain from *Chloroflexus aurantiacus* in a database search (UniProt Q3E5J3). Dawid et al. [20] have classified LIM domains into five groups. More recently, Kadrmas and Beckerle described only four distinct LIM groups and showed that invertebrates, like *Drosophila melanogaster* and *Caenorhabditis elegans* express nearly the same complement of LIM protein groups but show decreased complexity within each of them [21]. Both the PDZ and the LIM domains in proteins are most frequently found in combination with other domains. Most multi-domain proteins are related from gene fusions, deletions and internal repetitions [23]. An investigation of these evolutionary events requires a method to find the domain architecture from which each protein originates. The techniques of molecular phylogenetics, developed to recover the nested hierarchy of taxa from character information in their gene and/or protein sequences, can reconstruct the evolutionary family history. However, the evolutionary diversification of protein families often leads to structural differences, which makes their phylogenetic characterization difficult. Differences in domain architecture among multi-domain proteins for example often have raised the question of whether these proteins are orthologous, even though they have clearly arisen, at least in part, from a common ancestor. Considering all these problems, it has been suggested that the concept of orthology is applicable only at the level of domains rather than at the level of proteins [24], [25], except for proteins with identical domain architectures. Recently, the LAP family, which contain genes with both LRR and PDZ domains, has been classified by phylogenetic analysis showing the feasibility of an approach using domain sequences to obtain phylogenetic data [26].

The PDZ/LIM family is a good example of a multi domain protein family with diverse gene architectures. All family members have been shown to be able to associate with the actin cytoskeleton [e.g. 27], [28]–[30]. The ALP and Enigma subfamily genes are together with LMO7 able to bind α-actinin via their PDZ domains [e.g. 31], [32], [33]. Important biological roles have been described for muscle and heart development (ZASP [34], [35], ALP [36]), bone morphogenesis (Enigma [37]) and development of the nervous system and reproductive cells (LIM kinases [38]–[41]). In addition, LMO7, Mystique, RIL and the LIM kinases have all been linked to carcinogenesis and metastasis [42]–[47].

In order to characterize this family, we used a combinatorial approach, analyzing phylogenies of intronic sequences, of full length sequences and of sequence information for structural domains. Our results show that it is possible to derive a meaningful model for the molecular evolution of the PDZ/LIM family and characterize the phylogeny of its members.

## Results

### Genomic structures and gene architectures of the PDZ/LIM family

**Figure 1:**
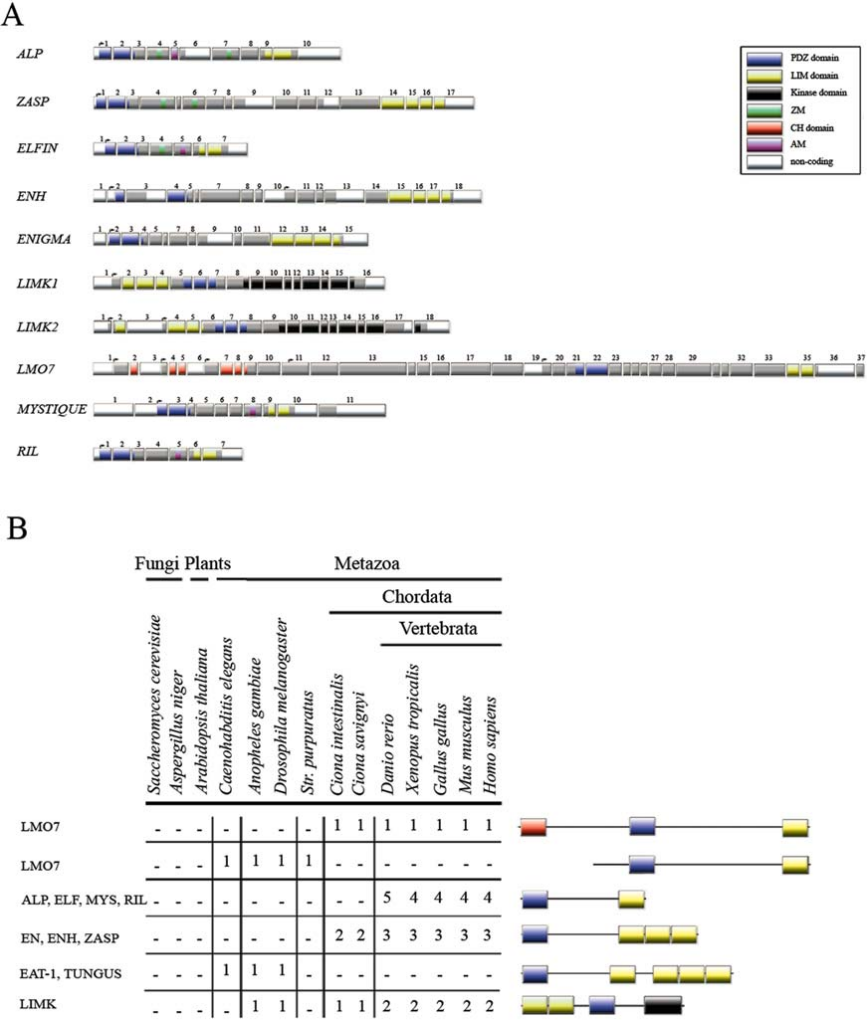
Exon structure, domain composition and the six basic forms of PDZ/LIM genes. (A) The exon composition of the human PDZ-LIM domain encoding genes in alphabetical order. Indicated are *ALP* (ENSG00000154553), *ZASP* (ENSG00000122367, Elfin (ENSG00000107438), ENIGMA (ENSG00000196923), *Enigma-Homolog* (ENH) (ENSG00000163110) *LIMK1* (ENSG00000106683, OTTHUMG00000023448), *LIMK2* (ENSG00000182541), *LMO7* (ENSG00000136153), *Mystique* (ENSG00000120913) and RIL (ENSG00000131435). Domains are color coded on the exons: LIM yellow, PDZ blue, CH red and ZM motif green, while transcription start sites are indicated after non coding regions (colored white) with a small arrow on top. (B) Presence of domain architectures for PDZ and LIM genes and their species distribution. Six basic gene structures can be found amongst the different taxons. The *tungus gene*, found in the two *arthropod species investigated and the nematode homolog Eat-1 both* encode one N-terminal PDZ and four C-terminal LIM domains. Eat-1 has been described earlier as the *Caenorhabditis elegans* ALP/Enigma gene [50]. Only a single *LIMK* gene was found per invertebrate species examined, and the *LMO7* homolog lacks the CH domain (*CG31534*). The LMO7 gene of *Drosophila* melanogaster lacks not only the CH domain but also the PDZ domain (not shown, see Supplemental table S1). As not all ALP and Enigma subfamily members share the ZM domain (ZASP and ALP contain 2 and Elfin one ZM motif) we have excluded the ZM motif from these groups and show only a ZM motif for eat-1/tungus in this figure.

An overview of the gene architectures for the ten human genes encoding the PDZ/LIM protein family is shown in figure 1A. All genes contain a single central- or N-terminal-positioned PDZ domain. Single or multiple LIM domains are positioned either N-terminal or C-terminal from the PDZ domain (Fig. 1B). Besides the PDZ and LIM domains, different motifs and domains can be found. Another protein interaction domain found in LMO7, is the Calponin homology (CH) domain [see for a review e.g. 48]. One catalytic domain, a tyrosine kinase domain is present in both LIMK1 and LIMK2. Furthermore, a ZASP-like motif (ZM motif) is found in *ZASP, ALP* and *Elfin* [49]. The ZM motif has been described to function in concert with the PDZ domain to localize ZASP to α-actinin, the major Z-disk cross linker in sarcomers [28], [49].

A close examination of the gene architecture of the PDZ and LIM domain encoding genes found in metazoan taxons reveals different combinations for the assembly of these functional domains (Fig. 1B). Four groups of combinations, representing LMO7, the ALP subfamily, the Enigma subfamily and the LIMKs respectively, are found in vertebrates. Both the number of combinations as well as the total number of genes found increases from the invertebrates to the vertebrates (see Fig. 1B). Only two different combinations can be found in C*aenorhabditis elegans*: *eat-1*, the previously described single gene “ALP/Enigma” homolog [50] and *tag204* (temporarily assigned gene 204), the* Caenorhabditis elegans LMO7* homolog. In *Drosophila melanogaster,* an *eat-1* homolog (*tungus*), a *LMO7* homolog (CG31534) and a *LIMK1* (AB042816) homolog is found. In contrast to both the *Caenorhabditis elegans* and the *Drosophila melanogaster LMO7* homologs, *LMO7* of *Ciona intestinalis* appears to have a CH domain. No combination of PDZ and LIM domain(s) was found for taxons in the *Plantae* or *Fungi* lineages with the BLAST algorithm [4] in the databases used (see Material and Methods).

### Evolution of the ALP/Enigma subfamilies

As an initial starting point to study the molecular evolution of the PDZ/LIM family, we performed the phylogenetic analysis of the ALP and Enigma subfamilies, using full length amino acid sequences for the different groups (see table S1 and S2 for accession numbers).

**Figure 2:**
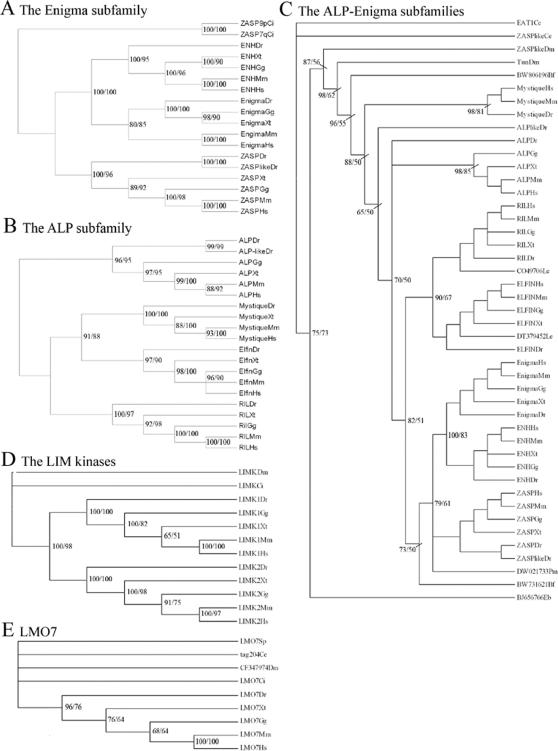
Phylogenetic trees of the Enigma and ALP subfamilies, the LIM kinases and LMO7 using full length sequences. (A,B,D and E) and PDZ domain sequences (C). In all phylogenetic trees shown, are the percentages for Bayesian posterior probability (first number) and for Maximum Likelihoods (second number) indicated at the branches. In figure C only the major branches are labeled for better overview. The two letter abbreviations used refer to genus and species, with the first capital letter and the second non-capital letter, respectively. All abbreviations used are given in material and methods. ENH is used for Enigma homolog (A) Shown is a phylogenic tree based on the full-length sequences of members of the Enigma subfamily. (B) Phylogenetic analysis for the ALP subfamily, based on full length sequences of selected homologs. (C) Evolutionary tree based on the PDZ domains of both ALP and Enigma subfamilies. (D) Phylogeny of the LIM domain kinases based on full length amino acid sequences. The root is placed on the *Drosophila melanogaster* homolog. The fruitfly homolog (which roots with the Uruchordate homolog found in *Ciona intestinalis*) gives rise to the common ancestor for the vertebrate LIM kinases. This ancestor then duplicates into LIMK1 and LIMK2, which are both present in all the vertebrate species investigated. (E) Evolutionary tree of LMO7 genes. The tree is rooted to the *Caenorhabditis elegans* sequence of tag-204, which has a similar structure to LMO7, albeit without the CH domain.

Figure 2A shows the rooted phylogenetic tree inferred for the Enigma subfamily using the *Ciona intestinalis* ZASP sequences as an outgroup. It shows a topology of the form (ZASP (Enigma, Enigma Homolog)). The ZASP homolog, with one PDZ and three LIM domains, was present in both urochordates and vertebrates (see Chordata in Fig. 1B). The phylogenetic tree in figure 2A clearly shows the split between urochordates and vertebrates.

The analysis of the ALP subfamily shows a topology of the form (ALP (RIL (Elfin, Mystique))) (Fig. 2B and Figure S1).

A combined phylogenetic analysis for the PDZ domain sequences of the two subfamilies is shown in figure 2C. The results here indicate that both the ALP subfamily and the Enigma subfamily evolved from a “one PDZ four LIM” ancestral gene like *eat-1/tungus* (tungus being the *Drosophila melanogaster* ortholog). The data suggest that loss of three LIM domains (LIM 2–4) from the common 4 LIM domain-containing ancestral gene leads to the ALP subfamily; whereas loss of one LIM domain (LIM1) leads to the three LIM domain containing Enigma subfamily. To further investigate the separation between the ALP and Enigma groups, we have searched for PDZ/LIM genes in *Ciona intestinalis* (Urochordata), in Amphioxus (Euchordata) in hagfish (Hyperotreti), in the lamprey (Hyperoartia) and in sharks and rays (Chondrichthyes). The results of the phylogenetic analysis are shown in figure 2C.

### Evolution of the LIM kinases and LMO-7

Similar to the limited approach, using full length sequences for only the subfamilies, we analyzed the LIM kinases and LMO7 (Fig. 2D and 2E, respectively). Figure 2D indicates a gene duplication event in the common ancestor for both LIM kinases, but also suggests that the separation leading to *LIMK1* and *LIMK2* did not occur before the chordates split from the other deuterostomes, since it appears that the ancestral LIM kinase gene duplicated after *Ciona intestinalis*. We further observed that the *LIMK2* homologs of both *Xenopus laevis* (Q8QHM0) and *Xenopus tropicalis* (ENSXETP00000009075) encode only one LIM domain (in contrast to the *Xenopus LIMK 1*, which contains two LIM domains), suggesting that the frogs lost a LIM (see table S1).

### Identification of novel ZASP-like genes containing a PDZ domain and a ZM motif

A short zebrafish ZASP-like gene containing only the PDZ domain and a ZM-motif had been described in GenBank (NM_201505). We described earlier similar structured short splice forms of the zebrafish *ZASP* gene, which could point to important functional similarities. In this study, we have now identified short ZASP-like genes in several species (*Caenorhabditis elegans, D. melanogaster, and Ciona intestinalis*) but were not able to find another vertebrate homolog in addition to the zebrafish gene. In an analysis based on the PDZ domain sequences (Fig. 2C) all invertebrate *ZASP-like* homologs segregated close to *eat*-1 and *tungus* whereas the zebrafish *ZASP-like* gene is closely related to zebrafish *ZASP*, indicating that a partial duplication event (or a complete duplication followed by partial truncation) occurred twice during evolution (Fig. 2C) and generated the ZASP-like structure. The zebrafish also contains an extra gene that resembles the normal members of the ALP subfamily. We have previously shown via a basic phylogenetic analysis on the full length amino acid sequence that the *alp-like* gene, clusters very closely to the normal *alp* gene of zebrafish [51]. In contrast to *alp, alp-like* lacks one of the ZM-motifs and is only found in zebrafish not in any other species including other fishes (Fig. 2C).

### Identification of a novel highly-conserved ALP-like motif (AM)

**Figure 3:**
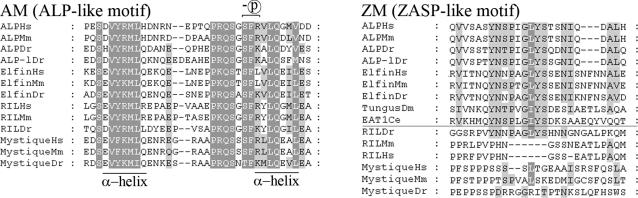
Sequence alignments showing the conserved motifs. (A) Conserved amino acids constituting the Alp-like (AM) motif, which was only present in the ALP family genes and neither found neither in the Enigma subfamily nor in their mutual precursor *eat-1/tungus.* (B) Conserved amino acids constituting the ZASP-like (ZM) motif found in both ALP subfamily and Enigma subfamily genes.

Examination of the multiple amino acid sequence alignments, between PDZ/LIM family proteins of different species revealed a novel motif, specific for the ALP subfamily members, which we denoted ALP-like motif (AM) (Fig. 3). The motif was not present in any known motif/structure databases. The primary 34 amino acid long sequence of the ALP-like motif contains a putative consensus PKC phosphorylation site and secondary structure prediction suggests two α-helices, one in the beginning and one at the end (Fig. 3). A closer look into the genomic structures of the *ALP* subfamily genes indicated that this domain was always encoded by the fifth exon. Together these findings suggest that the ALP-like motif must have evolved after the separation of the ALP and Enigma subfamilies.

This is in contrast to the ZASP-like motif (ZM) which is found in both subfamilies as well as in the ancestral Eat-1 gene. In figure 3 we show alignments for both motifs, however it is important to note that we have newly discovered the ALP-like motif and denoted it AM in analogy to the earlier denoted ZASP-like motif (ZM), but no structural or functional similarity is apparent between both motifs. Future studies are warranted to shed light on the function of the Alp-like motif (AM).

### Evolution of the PDZ and LIM domains

The phylogenetic analysis of specific individual domains, or their combinations, obtained from multiple domain-containing proteins can give insights into the mechanisms of protein evolution. Any phylogenetic analysis on full length sequence alignments may fail as robust structural variations, which are often present among protein groups or families, can prevent such an approach. The PDZ/LIM family illustrates complex domain arrangements in a multi-domain protein family (Fig. 1A and 1B).

We analyzed full length cDNA-, EST-derived- and genomic- PDZ and LIM domain sequences from over 25 species, ranging from yeast to humans (see table S1). All results obtained were supported by high Bayesian and Maximum likelihood support values.

**Figure 4:**
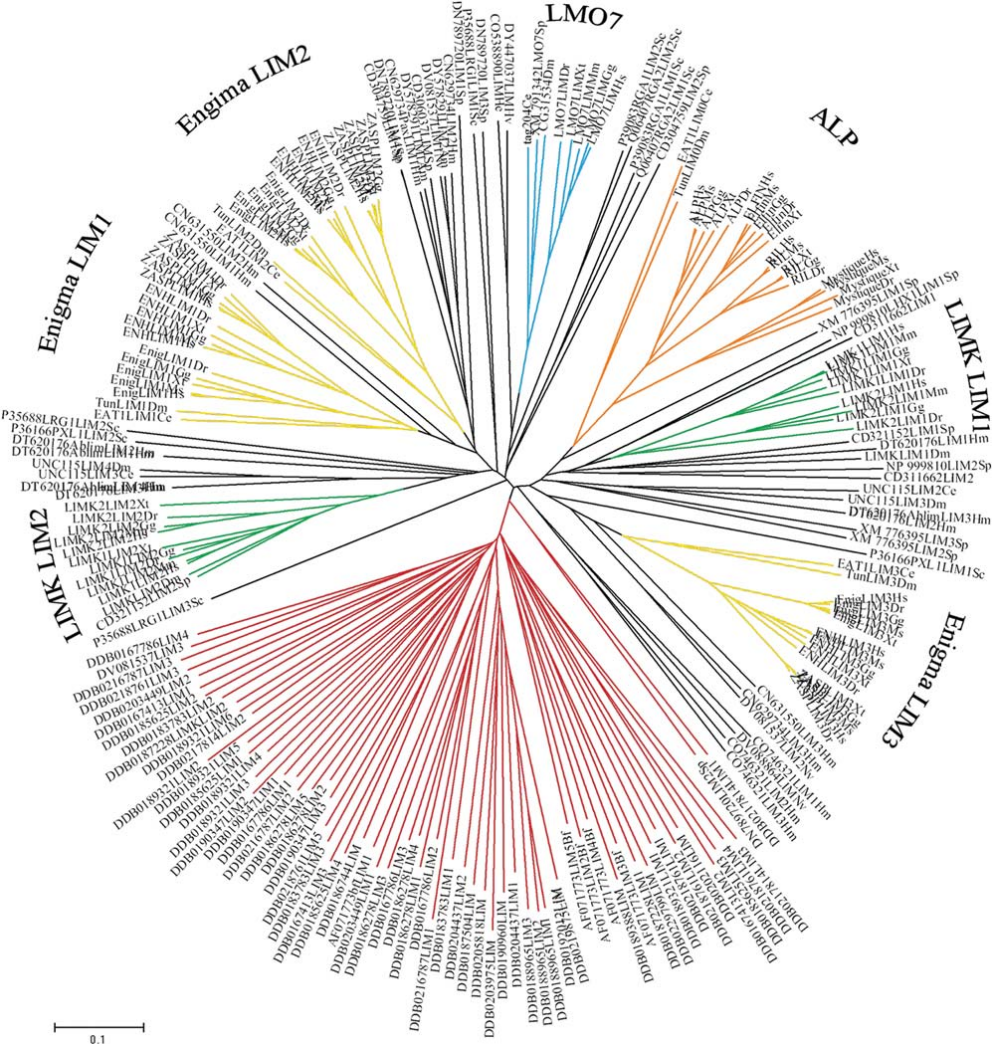
Dendogram of a representative set of LIM domains. Sequence comparisons of LIM domains in a Dendogram, with different clusters color-coded. We included all non-redundant LIM domains found in *Dictyostelium discoideum* and all from *C. elegans* and added the closest homologs of PDZ/LIM family LIM domains from different species, which were identified via BLAST search. The Dictyostelium discoideum sequences are marked in red and cluster all together and not with any other of the LIM domain clusters.

The initial dendogram derived from amino acid sequences of LIM domains (Fig. 4) illustrates the different clusters for the subfamilies and groups and individual LIM domains. The dendogram shows that three LIM domains of the Enigma subfamily individually cluster together (Fig. 4). It further demonstrates that all LIM domains found in the Protist *Dictyostelium discoideum* all cluster together (marked in red) to the exclusion of LIM domains in other species. Therefore, we used a *Dictyostelium discoideum* LIM domain as an outgroup for our phylogenetic analysis of the LIM domains.

**Figure 5:**
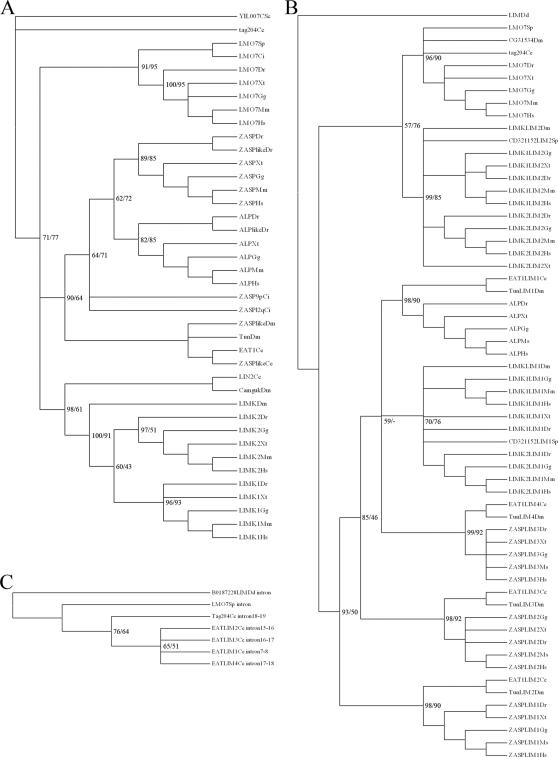
Phylogenetic analysis of PDZ and LIM domains. (A) The evolutionary tree for the PDZ domains is shown. The percentages for Bayesian posterior probability (first number) and for Maximum Likelihoods (second number) are indicated at the branches. The tree is rooted using the PDZ domain of the yeast (*S. cerevisiae*) NAS2 protein as an outgroup. The percentage Bayesian posterior probability and percentage Maximum Likelihood are indicated at the branches (Bayesian/Likelihood). All species can be identified from the last two letters of the taxon labels. All species cluster according to their appearance in the tree of life (www.tol.org) with the exception of the LIMK1 for *Xenopus tropicalis* and *Gallus gallus*, which are in reverse order, this was already seen in the full length analysis of the LIM kinases for the frog and chicken homolog (Fig. 2D). (B) The phylogenetic analysis of the LIM domains is shown. The tree is rooted using the LIM domain of the yeast (*S. cerevisiae*) LRG1 protein as an outgroup. Percentage Bayesian posterior probability and percentage Maximum Likelihood are indicated at the branches (Bayesian/Likelihood). (C) Shown is the phylogenetic analysis of conserved intronic-sequences of LIM domains of tag204, eat-1, LMO7 and a *Dictyostelium discoideum* LIM domain as an outgroup.

The evolutionary trees of the PDZ- and the LIM-domains, derived from this analysis are depicted in figure. 5A and 5B, respectively. Rooted evolutionary trees are shown (using yeast (Fig. 5A) and *Dictyostelium discoideum* (Fig. 5B) sequences as outgroups). For a better overview of the complete analysis, only ALP and ZASP are shown here as examples for the ALP- and Enigma-subfamilies.

The phylogenetic tree for the different PDZ domains shows that the most ancestral PDZ domain found in the family is LMO-7, with tag-204 being closest to the root and in front of all later clades. In a BLAST search, we identified the PDZ of CASK as the best aligning sequence to the LIMK PDZ and included sequences for the *Caenorhabditis elegans* (Lin2) and *Drosophila melanogaster* (Camguk) CASK homologs in the analysis. The PDZ phylogeny shows that both CASK homologs also relate to the “LMO7 common ancestor” and further show that their PDZ domain is closely related to the PDZ domain of the LIM kinases (Fig. 5A). Other MAGUK PDZ domains also showed that they originated from a “LMO7 common ancestor” (data not shown).

This suggests that the common ancestor of the LIM kinases and Lin2 and CASK lost the C-terminal LIM domain. Consequently, the PDZ domain of the “LMO7 common ancestor” is of central importance for the molecular evolution of the PDZ/LIM family.

The analysis of the LIM domains shows an early split between the LMO7 group and the LIM domain 2 of the LIMKs on one hand and all other LIM domains on the other hand. A PDZ single LIM structured common ancestor (as also seen for the PDZ phylogeny) is suggested by these results. We further examined a conserved intronic-sequence we have discovered in the LIM domains of *tag204* and *eat-1* (Fig. 5C).

**Figure 6:**
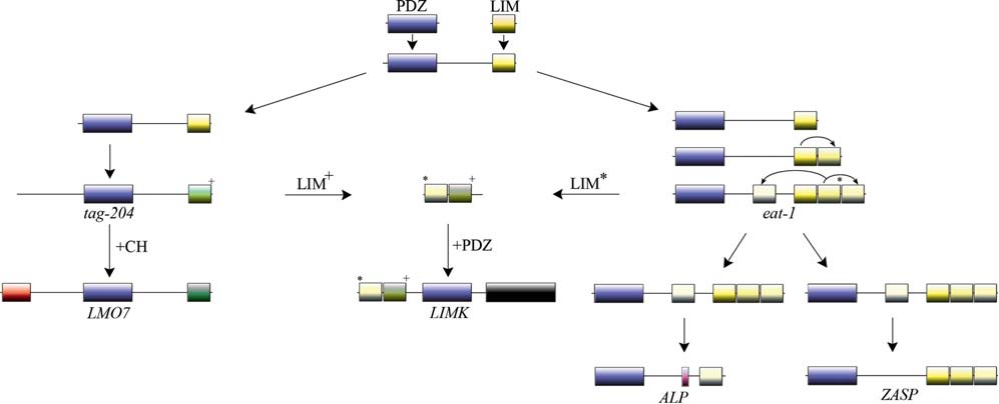
Evolutionary model for the PDZ and LIM encoding genes. The most parsimonious model derived from our phylogenetic analysis shows that the PDZ domain of all 10 different PDZ/LIM encoding genes share a common ancestor, with closest homology to LMO7. The combination of this PDZ domain with a LIM domain formed the common ancestor for both the LMO7 and the ALP/Enigma lineages. The single LIM domain in the ALP/Enigma lineage (closest to LIM2 in *eat-1/tungus)* then duplicated and gave rise to a PDZ two LIM domain structure. Subsequently the duplicated LIM (closest to LIM3 in *eat-1/tungus)*, duplicated twice and generated a PDZ four LIM structure similar to *eat-1/tungus*. From this gene structure, through gene duplication and subsequent domain loss (loosing either three LIM domains (LIM2–4) or only 1 LIM domain (LIM1) for the ALP and Enigma subfamilies, respectively), the ALP/Enigma subfamilies evolved. The color code used for domains is PDZ (blue), LIM (yellow and green), CH (red), Kinase domain (black) and AM-motif (pink).

The most parsimonious explanation derived from both the analysis of the PDZ domain tree and from the LIM domain tree is summarized in our model for the molecular evolution of the PDZ/LIM family (Fig. 6). The LIM domains of the LIMKs are related to the other PDZ/LIM family members (as is the PDZ domain); however gene rearrangements were necessary to generate the gene architecture of the LIMKs. A convergent event in respect to the combination of PDZ and LIM domains is indicated by our analysis, signifying that the PDZ has combined with the LIMs more than once during evolution. A single *LIMK* gene was identified in *Drosophila melanogaster*. No *LIMK* gene was found in *Caenorhabditis elegans* and lower taxons and only two PDZ/LIM genes are present in *Caenorhabditis elegans*: *tag20*4 and *eat-1*. We performed a BLAST search for the LIM domains closest to the LIMK LIMs in *Caenorhabditis elegans* and found UNC-115 which was also found in *Drosophila melanogaster*. Interestingly, the first two LIM domains of UNC-115 from *Caenorhabditis elegans* and *Drosophila melanogaster* cluster with the LIM kinase LIM 1 and 2 domains, suggesting that the two LIM domains were first assembled and then met the PDZ (see table S3 for sequence homologies). Taken together, our findings led us to propose a model which gives a plausible scenario for the molecular evolution of the PDZ/LIM genes and their diverse gene architecture (Fig. 6).

### Chromosomal location of ALP/Enigma subfamily genes

**Figure 7:**
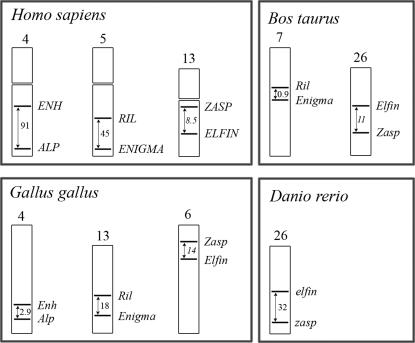
Chromosomal locations of ALP and Enigma subfamily genes. Shown are four species were PDZ/LIM genes are co-localized on the same chromosome. Numbers indicated are distances in mega basepairs (bp). If genes were found on individual chromosomes they are not shown here. Further not shown are the results for *Pan troglodytes* and *Macaca mulatta* which show exactly the same chromosomal distribution as observed for the humans. Not shown is also the data for *Rattus norwegicus*, where LIMK1 was co localized with LMO7, but no other combination was found. No combinations whatsoever were found in Mus musculus and in Canis familaris. It must be noted that for some of the species investigated the genomes are not completely sequenced and/or fully assembled.

As previously mentioned [50], the human ALP/Enigma genes cluster specifically in a way that an ALP-like gene pairs with an Enigma-like gene in inverse orientation, analogous to the even-skipped genes, which both (EVX1 and EVX2) are transcribed in an opposite orientation as compared to adjacent HOX genes [52]. This intuitively suggests a form of genome duplication as a plausible mechanism for their evolution. To further investigate this we looked at the chromosomal location of the ALP/Enigma genes in different species. ALP/Enigma clusters are observed in four different species indicating that this clustering appears more than random (Fig. 7). However, several species (mouse, dog) do not show any clustering, making these results difficult to interpret in an evolutionary context.

## Discussion

In this study, we have approached two fundamental problems in the study of molecular evolution. One is the “problem of comparison”, or how to compare very differently structured elements (many multi-domain proteins are only homologous along parts of their sequences); the other is the “problem of origin”, or how to determine which members in a complex family of proteins share a common ancestral gene. We have focused our analysis on the co-evolution of two conserved functional domains and have chosen to study the PDZ/LIM family for several reasons: the members of this family play important biological roles in development and for actin cytoskeleton organization, the entire family has never been analyzed before (and could not, in a conventional approach) and last, the diverse combinations of PDZ and LIM domains present in this family already suggested differences in their evolution.

We analyzed the phylogeny of the PDZ and the LIM domains separately, using a full length approach to analyze the individual subfamilies for a better resolution of later chains of events and looked at the distribution of PDZ/LIM genes in most sequenced eukaryotic lineages. The combined analysis and interpretation, and the merger of these results allowed us to classify the PDZ/LIM family and draw a plausible phylogenetic model.

According to our analysis, the PDZ/LIM gene family in mammals, as defined by a common ancestral gene, has 8 members, including *LMO7* and the 7 *ALP/Enigma* genes. This is supported by a comparison of a conserved intronic sequence situated in the LIM domains of the two *Caenorhabditis elegans* family members (Fig. 5C).

The PDZ and LIM domains of both the PDZ/LIM genes and the LIM Kinases have most likely evolved from the same ancestral domains. However, the fact that these domains have been combined twice during evolution is a convergent phenomenon. This convergent event, which we describe here for the domain combinations, might be of functional relevance. Indeed, all 10 PDZ/LIM genes have been shown to be able to associate with the actin cytoskeleton [27], [28], [31]. It is possible, that the combination of LIM and PDZ domains in a single functional module is necessary for specific interactions with both the actin cytoskeleton and other proteins. Thus, this could indicate a functional convergence for all 10 genes in organizing protein complexes associated with the actin cytoskeleton.

The *Caenorhabditis elegans eat-1* gene is related to the *Drosophila melanogaster tungus* gene and both share the same gene architecture, with one PDZ domain, a single ZM motif and four LIM domains. Both, PDZ domain and LIM domain phylogenies show that the ALP as well as the Enigma subfamily genes originate from eat-1/tungus like ancestor and separated late in evolution using the same PDZ encoding exons, but losing either the last three LIM encoding exons or the first, respectively (Fig. 2C and 5B). Here the LIM domains of the genes in the ALP family all group together with the first LIM domain of *eat-1* (we named it LIM1 in the tree), while the three LIM domains of the Enigma family segregate together with the last three LIM domains encoded by *eat-1*. This was already postulated by McKeown and colleagues when they functionally described the *eat-1* gene [50] and we confirmed this here in our analysis with high support values. Looking at a smaller evolutionary window, we have evidence that the separation occurred between the Euchordata and the early vertebrata with a ZASP homolog found in amphioxus and lamprey, and RIL and Elfin homologs found in an early vertebrate, like the ray (Chondrichthyes) (Fig. 2C).

The basic model for the evolution of a multi-domain protein family and the original definition of this gene family (defined by a common ancestral gene) is suggested with high likelihood by our findings. Our phylogenetic interpretation of the evolution of the PDZ/LIM family shows that the LIMKs are the most distantly related genes, whereas all others, including LMO7, appear to have a common ancestral gene and thus constitute a classical gene family.

## Materials and Methods

### Creating a functional domain database

First a dataset of all functional domains of all PDZ and LIM domain encoding genes was established. For this purpose human protein sequences were BLASTed [4] against the genome and EST databases of Ensembl (http://www.ensembl.org/index.html), National Centre for Biotechnology Information (http://www.ncbi.nlm.nih.gov/) and the Joint Genome Institute (http://www.jgi.doe.gov/). Blast hits with high enough E-values were further investigated with the protein domain prediction program SMART [53]–[55]. Homologs were searched for in the following species: *Anopheles gambiae*, *Arabidopsis thaliana*, *Bos taurus*, *Danio rerio* (Dr), *Caenorhabditis elegans* (Ce), *Canis familiaris*, *Ciona intestinalis* (Ci), *Ciona savignyi*, *Drosophila melanogaster* (Dm), *Gallus gallus* (Gg), *Homo sapiens* (Hs), *Mus musculus* (Mm), *Pan troglodytes*, *Phytophtora sojae*, *Populus trichocarpa*, *Saccharomyses cerevisiae* (Sc), *Strongylocentrotus purpuratus* (Sp), *Takifugu rubripes*, *Tetraodon nigroviridis*, *Xenopus laevis* and *Xenopus tropicalis* (Xt). In our phylogenetic analysis however, we have excluded several species (e.g. some of the mammals) as their inclusion did not add any significant value for inferring an evolutionary model.

### Alignment and phylogenetic analysis

Alignments were performed using ClustalX [56] with default parameter values, and manually refined in GeneDoc where necessary. Reliably aligned regions in full length alignments were selected with Gblocks [57]. The minimum length for conserved blocks was set to five residues, while we decided to keep gap containing positions if the gap was present in 50% of the sequences examined. It has to be noted here that even under less restricted conditions, Gblocks selected only the domains for analysis, which makes the number of characters used in the phylogenetic analysis in the same order of magnitude as our domain specific analysis. The edited alignments were used for phylogenetic analysis employing both Bayesian analysis and maximum likelihood (ML). Bayesian trees were generated with MrBayes [58], with amino acid substitution set to mixed (hence reducing assumption prior to analysis). Rate variation across sites was modeled with a four rate gamma distribution and invariant sites, while the MCMC search itself was continued for 1.000.000 generations, sampled every 100 generations, and 2500 trees were discarded as burnin. For ML, alignments were bootstrapped 1,000 times with the program SEQBOOT from the PHYLIP package [59]. Subsequently, phylogenetic trees were generated with the ML algorithm implemented in PHYML [60], whereas a consensus tree was calculated with Consense from the PHYLIP package [59]. Parameters for PHYML were set at Jones-Taylor-Thornton for amino acid substitution and gamma distribution with four classes for across-site rate variation. The alpha parameter of the gamma distribution was estimated by PHYML. At last, phylogenetic trees were visualized with either NJplot [61] or MEGA 3.1 [62]. In the tree figures shown, the topology support values are labeled on the Bayesian consensus tree in the order % Bayesian posterior probability/ % bootstrap ML to reduce and standardize the characters and figures used.

## Supporting Information

Figure S1 Phylogeny by structures consisting of one PDZ and one LIM domain. Numbers indicate % Bayesian posterior probability. Sequences used are specified in supplemental table S1, with the exception of the EAT splice form 1A (consisting of 1 PDZ and 1 LIM domain) for which the acc. number CAE52906 was used.
(0.68 MB TIF)

Table S1 DNA and amino acid sequences of protein interaction domains studied. All accession numbers used are listed.
(0.51 MB XLS)

Table S2 List of PDZ and LIM sequences used in this study.
(0.09 MB DOC)

Table S3 Obtained BLAST results for assorted PDZ and LIM domains.
(0.06 MB DOC)

## References

[1] Hergyi H, Gerstein M (1999). The relationship between protein structure and function: a comprehensive survey with application to the yeast genome.. Journal of Molecular Evolution.

[2] Apic G, Gough J, Teichmann SA (2001). An insight into domain combinations.. Bioinformatics.

[3] Copley RR, Doerks T, Letunic I, Bork P (2002). Protein domain analysis in the era of complete genomes.. FEBS Letters.

[4] Altschul SF, Madden TL, Schaffer AA, Zhang J, Zhang Z (1997). Gapped BLAST and PSI-BLAST: a new generation of protein database search programs.. Nucl Acids Res.

[5] Eddy SR (1996). Hidden Markov models.. Current Opinion in Structural Biology.

[6] Chothia C (1992). One thousand families for the molecular biologist.. Nature.

[7] Yuri I, Wolf GKEVK (2002). Scale-free networks in biology: new insights into the fundamentals of evolution?. BioEssays.

[8] Hegyi H, Gerstein M (1999). The relationship between protein structure and function: a comprehensive survey with application to the yeast genome.. Journal of Molecular Biology.

[9] Henikoff S, Greene EA, Pietrokovski S, Bork P, Attwood TK (1997). Gene Families: The Taxonomy of Protein Paralogs and Chimeras.. Science.

[10] Gough J (2005). Convergent evolution of domain architectures (is rare).. Bioinformatics.

[11] Cho K-O, Hunt CA, Kennedy MB (1992). The rat brain postsynaptic density fraction contains a homolog of the drosophila discs-large tumor suppressor protein.. Neuron.

[12] Woods DF, Bryant PJ (1991). The discs-large tumor suppressor gene of Drosophila encodes a guanylate kinase homolog localized at septate junctions.. Cell.

[13] Itoh M, Nagafuchi A, Yonemura S, Kitani-Yasuda T, Tsukita S (1993). The 220-kD protein colocalizing with cadherins in non-epithelial cells is identical to ZO-1, a tight junction-associated protein in epithelial cells: cDNA cloning and immunoelectron microscopy.. J Cell Biol.

[14] Harris BZ, Lim WA (2001). Mechanism and role of PDZ domains in signaling complex assembly.. J Cell Sci.

[15] Ponting CP (1997). Evidence for PDZ domains in bacteria, yeast, and plants.. Protein Sci.

[16] Ponting CP, Phillips C, Davies KE, Blake DJ (1997). PDZ domains: targeting signalling molecules to sub-membranous sites.. Bioessays.

[17] Jelen F, Oleksy A, Smietana K, Otlewski J (2003). PDZ domains - common players in the cell signaling.. Acta Biochim Pol.

[18] Pallen MJ, Ponting CP (1997). PDZ domains in bacterial proteins.. Molecular Microbiology.

[19] Bach I (2000). The LIM domain: regulation by association.. Mech Dev.

[20] Dawid IB, Breen JJ, Toyama R (1998). LIM domains: multiple roles as adapters and functional modifiers in protein interactions.. Trends in Genetics.

[21] Kadrmas JL, Beckerle MC (2004). THE LIM DOMAIN: FROM THE CYTOSKELETON TO THE NUCLEUS.. Nature Reviews Molecular Cell Biology.

[22] Briere C, Bordel A-C, Barthou H, Jauneau A, Steinmetz A (2003). Is the LIM-domain Protein HaWLIM1 Associated with Cortical Microtubules in Sunflower Protoplasts?. Plant Cell Physiol.

[23] Thornton JW, DeSalle R (2000). Gene family evolution and homology: Genomics Meets Phylogenetics.. Annual Review of Genomics and Human Genetics.

[24] Koonin EV, Aravind L, Kondrashov AS (2000). The Impact of Comparative Genomics on Our Understanding of Evolution.. Cell.

[25] Ponting CP, Russell RR (2002). The natural history of protein domains.. Annual Review of Biophysics and Biomolecular Structure.

[26] Santoni M-J, Pontarotti P, Birnbaum D, Borg J-P (2002). The LAP family: a phylogenetic point of view.. Trends in Genetics.

[27] Yang N, Higuchi O, Ohashi K, Nagata K, Wada A (1998). Cofilin phosphorylation by LIM-kinase 1 and its role in Rac-mediated actin reorganization.. Nature.

[28] Klaavuniemi T, Ylanne J (2006). Zasp/Cypher internal ZM-motif containing fragments are sufficient to co-localize with α-actinin—Analysis of patient mutations.. Experimental Cell Research.

[29] Vallenius T, Luukko K, Makela TP (2000). CLP-36 PDZ-LIM Protein Associates with Nonmuscle alpha -Actinin-1 and alpha -Actinin-4.. J Biol Chem.

[30] Nakagawa N, Hoshijima M, Oyasu M, Saito N, Tanizawa K (2000). ENH, Containing PDZ and LIM Domains, Heart/Skeletal Muscle-Specific Protein, Associates with Cytoskeletal Proteins through the PDZ Domain.. Biochemical and Biophysical Research Communications.

[31] Ooshio T, Irie K, Morimoto K, Fukuhara A, Imai T (2004). Involvement of LMO7 in the association of two cell-cell adhesion molecules, nectin and E-cadherin, through afadin and alpha-actinin in epithelial cells.. Journal of Biological Chemistry.

[32] Andersen O, Ostbye TK, Gabestad I, Nielsen C, Bardal T (2004). Molecular characterization of a PDZ-LIM protein in Atlantic salmon (Salmo salar): a fish ortholog of the alpha-actinin-associated LIM-protein (ALP).. Journal of Muscle Research and Cell Motility.

[33] Zhou Q, Ruiz-Lozano P, Martone ME, Chen J (1999). Cypher, a striated muscle-restricted PDZ and LIM domain-containing protein, binds to alpha-actinin-2 and protein kinase C.. Journal of Biological Chemistry.

[34] Zhou Q, Chu P-H, Huang C, Cheng C-F, Martone ME (2001). Ablation of Cypher, a PDZ-LIM domain Z-line protein, causes a severe form of congenital myopathy.. J Cell Biol.

[35] van der Meer DLM, Marques IJ, Leito JTD, Besser J, Bakkers J (2006). Zebrafish cypher is important for somite formation and heart development.. Developmental Biology.

[36] Pashmforoush M, Pomies P, Peterson KL, Kubalak S, Ross J (2001). Adult mice deficient in actinin-associated LIM-domain protein reveal a developmental pathway for right ventricular cardiomyopathy.. Nat Med.

[37] Boden SD, Liu Y, Hair GA, Helms JA, Hu D (1998). LMP-1, A LIM-Domain Protein, Mediates BMP-6 Effects on Bone Formation.. Endocrinology.

[38] Takahashi T, Aoki S, Nakamura T, Koshimizu U, Matsumoto K (1997). Xenopus LIM motif-containing protein kinase, Xlimk1, is expressed in the developing head structure of the embryo.. Developmental Dynamics.

[39] Foletta VC, Moussi N, Sarmiere PD, Bamburg JR, Bernard O (2004). LIM kinase 1, a key regulator of actin dynamics, is widely expressed in embryonic and adult tissues.. Experimental Cell Research.

[40] Takahashi H, Koshimizu U, Miyazaki J-i, Nakamura T (2002). Impaired Spermatogenic Ability of Testicular Germ Cells in Mice Deficient in the LIM-Kinase 2 Gene.. Developmental Biology.

[41] Takahashi T, Koshimizu U, Abe H, Obinata T, Nakamura T (2001). Functional involvement of Xenopus LIM kinases in progression of oocyte maturation.. Developmental Biology.

[42] Kang S, Xu H, Duan X, Liu J-J, He Z (2000). PCD1, a Novel Gene Containing PDZ and LIM Domains, Is Overexpressed in Several Human Cancers.. Cancer Res.

[43] Loughran G, Healy NC, Kiely PA, Huigsloot M, Kedersha NL (2005). Mystique is a new insulin-like growth factor-I-regulated PDZ-LIM domain protein that promotes cell attachment and migration and suppresses Anchorage-independent growth.. Mol Biol Cell.

[44] Yoshioka K, Foletta V, Bernard O, Itoh K (2003). A role for LIM kinase in cancer invasion.. PNAS.

[45] Kiess M, Scharm B, Aguzzi A, Hajnal A, Klemenz R (1995). Expression of ril, a novel LIM domain gene, is down-regulated in Hras-transformed cells and restored in phenotypic revertants.. Oncogene.

[46] Bagheri-Yarmand R, Mazumdar A, Sahin AA, Kumar R (2006). LIM kinase 1 increases tumor metastasis of human breast cancer cells via regulation of the urokinase-type plasminogen activator system.. International Journal of Cancer.

[47] Suyama E, Wadhwa R, Kawasaki H, Yaguchi T, Kaul SC (2004). LIM kinase-2 targeting as a possible anti-metastasis therapy.. The Journal of Gene Medicine.

[48] Lehman W, Craig R, Kendrick-Jones J, Sutherland-Smith A (2004). An open or closed case for the conformation of calponin homology domains on F-actin?. Journal of Muscle Research and Cell Motility.

[49] Klaavuniemi T, Kelloniemi A, Ylanne J (2004). The ZASP-like Motif in Actinin-associated LIM Protein Is Required for Interaction with the {alpha}-Actinin Rod and for Targeting to the Muscle Z-line.. J Biol Chem.

[50] McKeown CR, Hand H-F, Berckerle MC (2006). Molecular characterization of the *Caenorhabditis* elegans ALP/Enigma gene alp-1.. Developmental Dynamics.

[51] te Velthuis AJW, Ott EB, Marques IJ, Bagowski CP (2007). Gene expression patterns of the ALP family during zebrafish development.. Gene Expression Patterns.

[52] A Faiella MDE, Rambaldi M, Acampora D, Balsofiore S, Stornaiuolo A, Mallamaci A, Migliaccio E, Gulisano M, Simeone A (1991). Isolation and mapping of EVX1, a human homeobox gene homologous to even-skipped, localized at the 5′ end of HOX1 locus on chromosome 7.. Nucleic Acids Research.

[53] Letunic I, Copley RR, Pils B, Pinkert S, Schultz J (2006). SMART 5: domains in the context of genomes and networks.. Nucleic Acids Research.

[54] Schultz J, Copley RR, Doerks T, Ponting CP, Bork P (2000). SMART: a web-based tool for the study of genetically mobile domains.. Nucleic Acids Research.

[55] Schultz J, Milpetz F, Bork P, Ponting CP (1998). SMART, a simple modular architecture research tool: identification of signaling domains.. Proceedings of the National Academy of Sciences of the United States of America.

[56] Thompson JD, Gibson TJ, Plewniak F, Jeanmougin F, Higgins DG (1997). The CLUSTAL_X windows interface: flexible strategies for multiple sequence alignment aided by quality analysis tools.. Nucleic Acids Research.

[57] Castresana J (2000). Selection of Conserved Blocks from Multiple Alignments for Their Use in Phylogenetic Analysis.. Mol Biol Evol.

[58] Huelsenbeck JP, Ronquist F (2001). MRBAYES: Bayesian inference of phylogenetic trees.. Bioinformatics.

[59] Felsenstein J (1989). PHYLIP – Phylogeny Inference Package (Version 3.2).. Cladistics.

[60] Guindon Sp, Gascuel O (2003). A Simple, Fast, and Accurate Algorithm to Estimate Large Phylogenies by Maximum Likelihood.. Systematic Biology.

[61] Perriere G, Gouy M (1996). WWW-query: An on-line retrieval system for biological sequence banks.. Biochimie.

[62] Kumar S, Tamura K, Nei M (2004). MEGA3: Integrated Software for Molecular Evolutionary Genetics Analysis and Sequence Alignment.. Briefings in Bioinformatics.

