# Supplementary figures and images for: Insights into the Molecular Evolution of the PDZ/LIM Family and Identification of a Novel Conserved Protein Motif
Source: PLoS One. 2007 Feb 7;2(2):e189. doi: 10.1371/journal.pone.0000189 (PMC1781342; doi:10.1371/journal.pone.0000189)

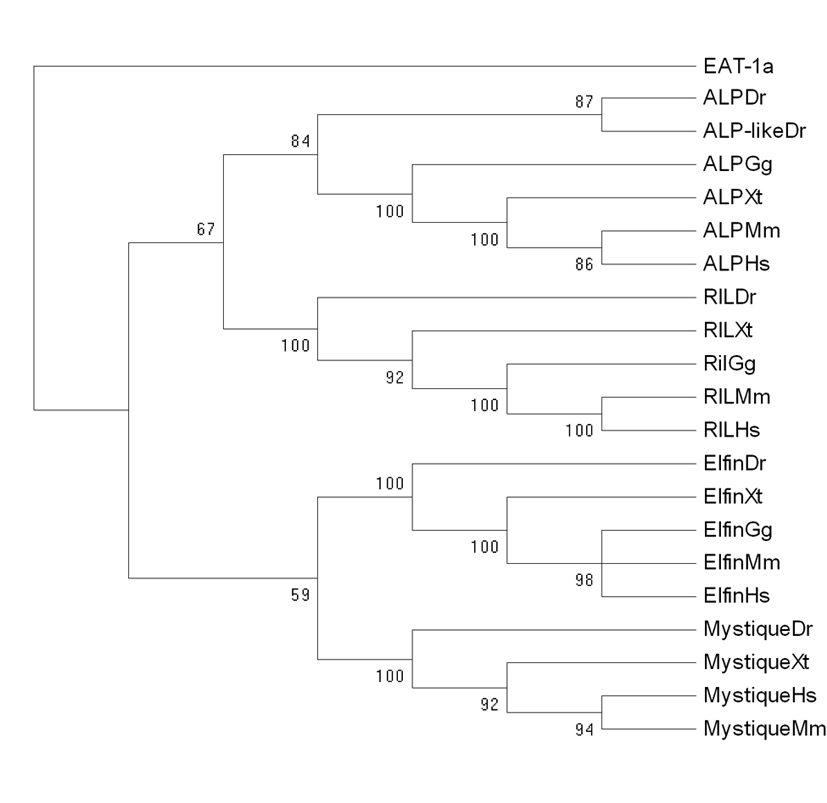

Supplement: Figure S1 — Phylogeny by structures consisting of one PDZ and one LIM domain. Numbers indicate % Bayesian posterior probability. Sequences used are specified in supplemental table S1, with the exception of the EAT splice form 1A (consisting of 1 PDZ and 1 LIM domain) for which the acc. number CAE52906 was used. (0.68 MB TIF) [file pone.0000189.s001.tif]
