# Supplementary material for: Insights into the Molecular Evolution of the PDZ/LIM Family and Identification of a Novel Conserved Protein Motif
Source: PLoS One. 2007 Feb 7;2(2):e189. doi: 10.1371/journal.pone.0000189 (PMC1781342; doi:10.1371/journal.pone.0000189)
Supplement: Table S2 — List of PDZ and LIM sequences used in this study. (0.09 MB DOC) [file pone.0000189.s003.doc]

Supplemental table 2: List of the PDZ and LIM sequences used in this study

| **Species** | **Accession numbers** | **Protein names** |
| --- | --- | --- |
| *Homo sapiens* | P53667 | LIMK1 |
|  | P53671 | LIMK2 |
|  | AAL37480 | LMO7 |
|  | AF039018 | ALP |
|  | U90878 | Elfin |
|  | BC021556 | Mystique |
|  | AF153882 | RIL |
|  | L35240 | Enigma |
|  | AF061258 | ENH |
|  | AJ133767 | ZASP |
| *Mus musculus* | P53668 | LIMK1 |
|  | O54785 | LIMK2 |
|  | BC049164, AK137694 | LMO7 |
|  | BC070418 | ALP |
|  | BC004809 | Elfin |
|  | BC024556 | Mystique |
|  | BC030068 | RIL |
|  | BC052698 | Enigma |
|  | BC037476 | ENH |
|  | AY206013 | ZASP |
| *Gallus gallus* | Q8QFP8 | LIMK1 |
|  | P53666 | LIMK2 |
|  | CR523526 | LMO7 |
|  | AJ249218 | ALP |
|  | CR387509 | Elfin |
|  | NM_204839 | RIL |
|  | AY376690 | Enigma |
|  | NM_001031149 | ENH |
|  | XM_421495 | ZASP |
| *X. tropicalis* | ENSXETP00000022723 ^*^ | LIMK1 |
|  | ENSXETP00000009075 ^*^ | LIMK2 |
|  | CX327108, CX327390 ^++^ | LMO7 |
|  | BC075363 | ALP |
|  | BC061431 | Elfin |
|  | CX995785 ^++^ | Mystique |
|  | BC063900 | RIL |
|  | BC091603 | Enigma |
|  | BC090094 | ENH |
|  | BC062490 | ZASP |
| *Danio rerio* | DQ679229 | LIMK1 |
|  | Q6DG29 | LIMK2 |
|  | CF347974 ^++^ | LMO7 |
|  | DQ679228 | ALP |
|  | BC095249 | ALP-like |
|  | BC092978 | Elfin |
|  | DQ679227 | RIL |
|  | DQ679230 | Mystique |
|  | BC061704 | Enigma |
|  | BC076551 | ENH |
|  | DQ012167 | ZASP |
|  | NM_201505 | ZASP-like |
| *C. intestinalis* | ENSCING00000002916 | LIMK |
|  | ENSCING00000001236, BW502563 ^++^ | LMO7 |
|  | ENSCING00000001795 ^*^ | ZASP (7q) |
|  | ENSCINP00000013859 ^*^ | ZASP (9p) |
|  | ENSCING00000007473 ^*^ | ZASP-like (2q) |
|  | ENSCING00000002452 ^*^ | ZASP-like (14q) |
| *D. melanogaster* | Q8IR79 | LIMK |
|  | NM_141231 | CG31534 |
|  | [NM_001043086](http://www.ncbi.nlm.nih.gov/entrez/viewer.fcgi?db=nucleotide&val=116007705) | Tungus |
|  | NM_139989 | ZASP-like |
|  | Q24210 | Camguk |
|  | Q8INN5 | Unc-115 |
| *C. elegans* | NM_171173 | tag-204 |
|  | [NM_001047522](http://www.ncbi.nlm.nih.gov/entrez/viewer.fcgi?db=nucleotide&val=115532933) | eat-1 |
|  | Q9XTP9, EC006763 ^++^ | ZASP-like |
|  | P54936 | LIN-2 |
|  | Q95QM5 | Unc-115 |
| *S. cerevisiae* | AY558294 | YIL007C |
|  | CAA55210 | LRG-1 |
|  | X90950 | Rga-1 |

^++^EST clones

^*^ Ensemble database accession number confirmed with EST sequences
