# Supplementary material for: Insights into the Molecular Evolution of the PDZ/LIM Family and Identification of a Novel Conserved Protein Motif
Source: PLoS One. 2007 Feb 7;2(2):e189. doi: 10.1371/journal.pone.0000189 (PMC1781342; doi:10.1371/journal.pone.0000189)
Supplement: Table S3 — Obtained BLAST results for assorted PDZ and LIM domains. (0.06 MB DOC) [file pone.0000189.s004.doc]

Supplemental table 3

| **Species** | **2^nd^ Species** | **Gene** | **Blast Query** | **Blast Hit** ^+^ | **Expect** | **% ID** |
| --- | --- | --- | --- | --- | --- | --- |
| *Drosophila* | *Drosophila* | *LIMK* | PDZ | Camguk ^#^ | 0.053 | 50% |
|  |  |  | LIM1 | Unc-115 LIM3 ^*^ | 6e-05 | 39% |
|  |  |  | LIM2 | Unc-115 LIM4 ^*^ | 2e-04 | 46% |
| *Drosophila* | *C. elegans* | *LIMK* | PDZ | LIN-2 ^#^ | 0.065 | 46% |
|  |  |  | LIM1 | Unc-115 LIM2 ^*^ | 3e-04 | 38% |
|  |  |  | LIM2 | Unc-115 LIM3 ^*^ | 4e-06 | 50% |
| *Drosophila* | *S. cerevisiae* | *LIMK* | PDZ | - | - | - |
|  |  |  | LIM1 | Rga1 LIM2 | 0.041 | 35% |
|  |  |  | LIM2 | DBY874  LIM1 | 0.46 | 26% |
| *C. elegans* | *C. elegans* | *Tag-204* | PDZ | ZK849.2/ C45G9.7 | 0.010/ 0.25 | 29%/ 45% |
|  |  |  | LIM | ZC64.4 | 0.001 | 33% |
| *C. elegans* | *S. cerevisiae* | *Tag-204* | PDZ | YIL007C | 1.3 | 45% |
|  |  |  | LIM | LRG1  LIM1 | 1.0 | 30% |
| *C. elegans* | *C. elegans* | *EAT-1* | PDZ | C45G9.7 | 0.19 | 36% |
|  |  |  | LIM0 | ZC64.4 | 0.037 | 51% |
|  |  |  | LIM1 | tag-327 LIM1 | 2e-05 | 41% |
|  |  |  | LIM2 | tag-327 LIM3/ 2 | 4e-10/ 7e-09 | 45%/ 38% |
|  |  |  | LIM3 | Tag-327 LIM3/ 2 | 4e-07/ 2e-06 | 40%/42% |
| *C. elegans* | *S. cerevisiae* | *EAT-1* | PDZ | - | - | - |
|  |  |  | LIM0 | - | - | - |
|  |  |  | LIM1 | - | - | - |
|  |  |  | LIM2 | LRG1 LIM1/ 2 | 3e-04/ 4e-04 | 46%/ 40% |
|  |  |  | LIM3 | LRG1 LIM1/ 2 | 0.27/ 1.7 | 40%/27% |

^+^ The closest BLAST-hits are shown other than the protein the blasted domain was used from. Also indicated are the corresponding Expect values and the percentage ID scores. The domains of these BLAST results were used to link the four major PDZ/LIM subfamilies together in one phylogenetic three.

^*^ The *Caenorhabditis elegans* Unc-115 holds 3 LIM domains while the *D. melanogaster* homolog holds the sequence for 4 LIM domains.

^#^ LIN-2 and Camguk both belong to the MAGI family and hold beside a PDZ domain, a SH3 and a GukC domain.
